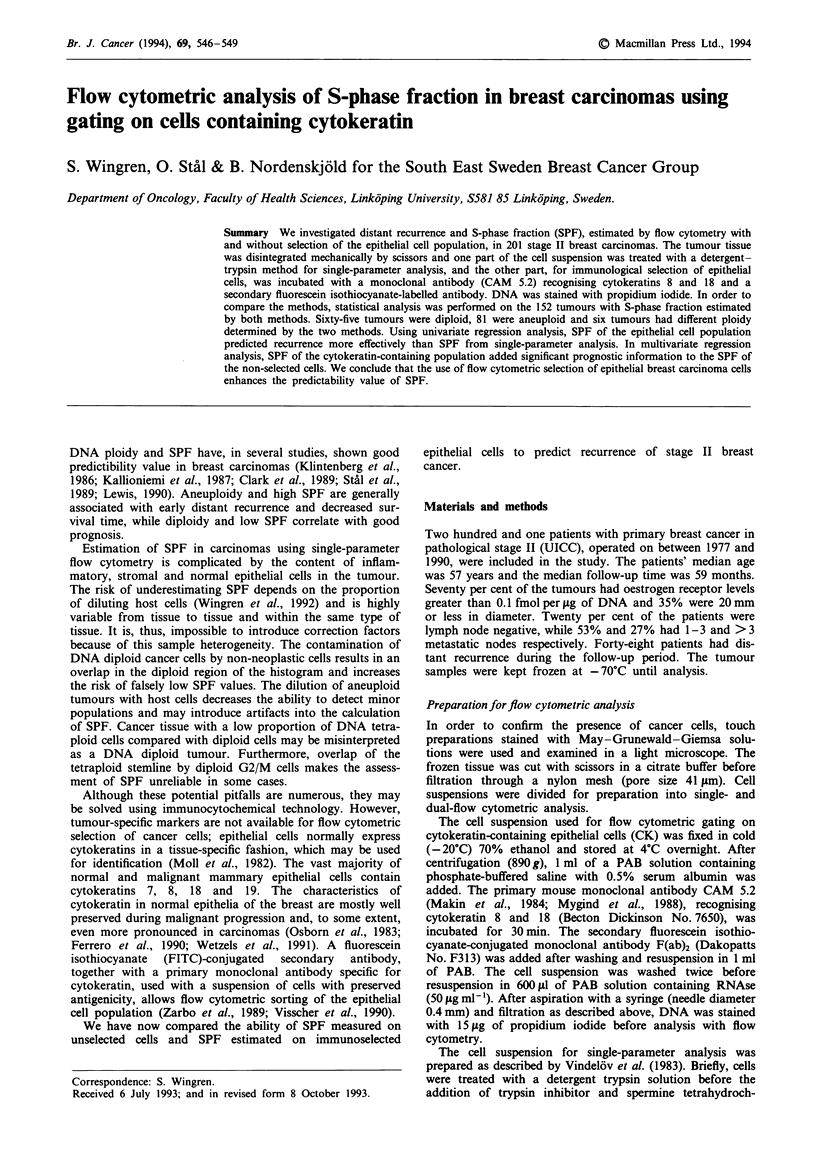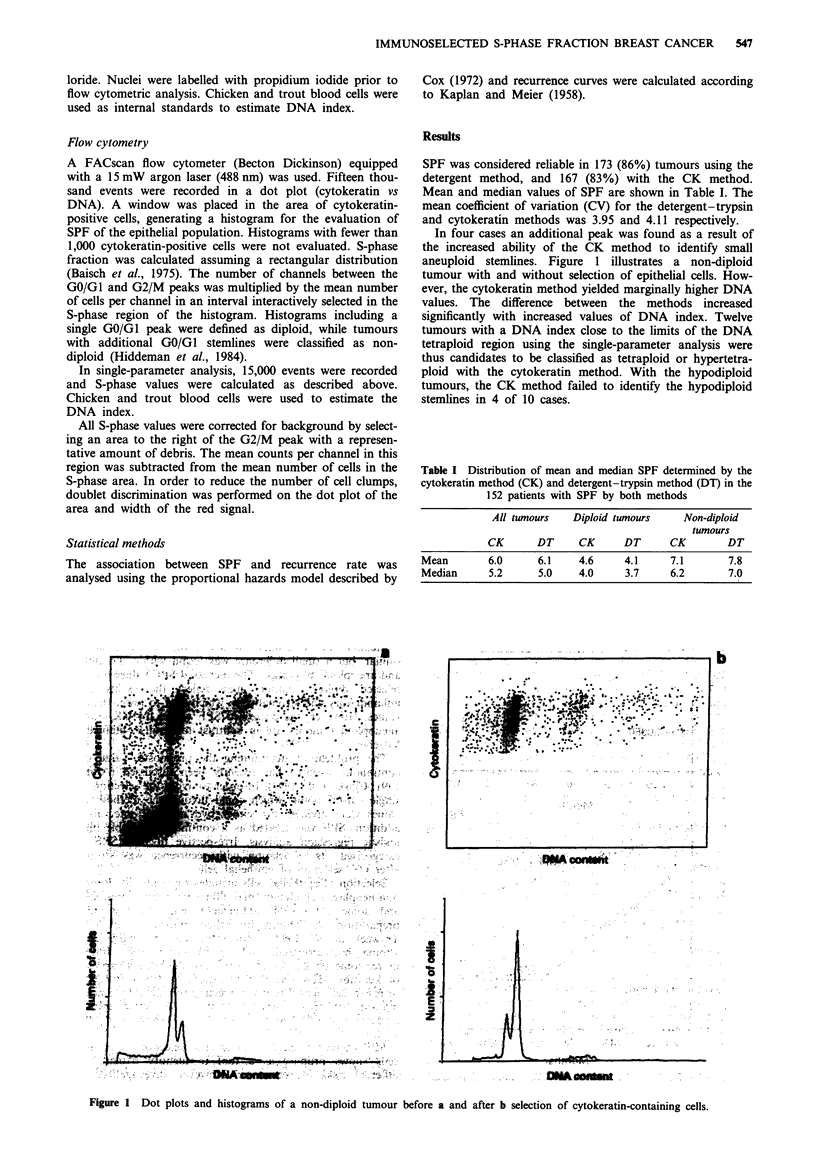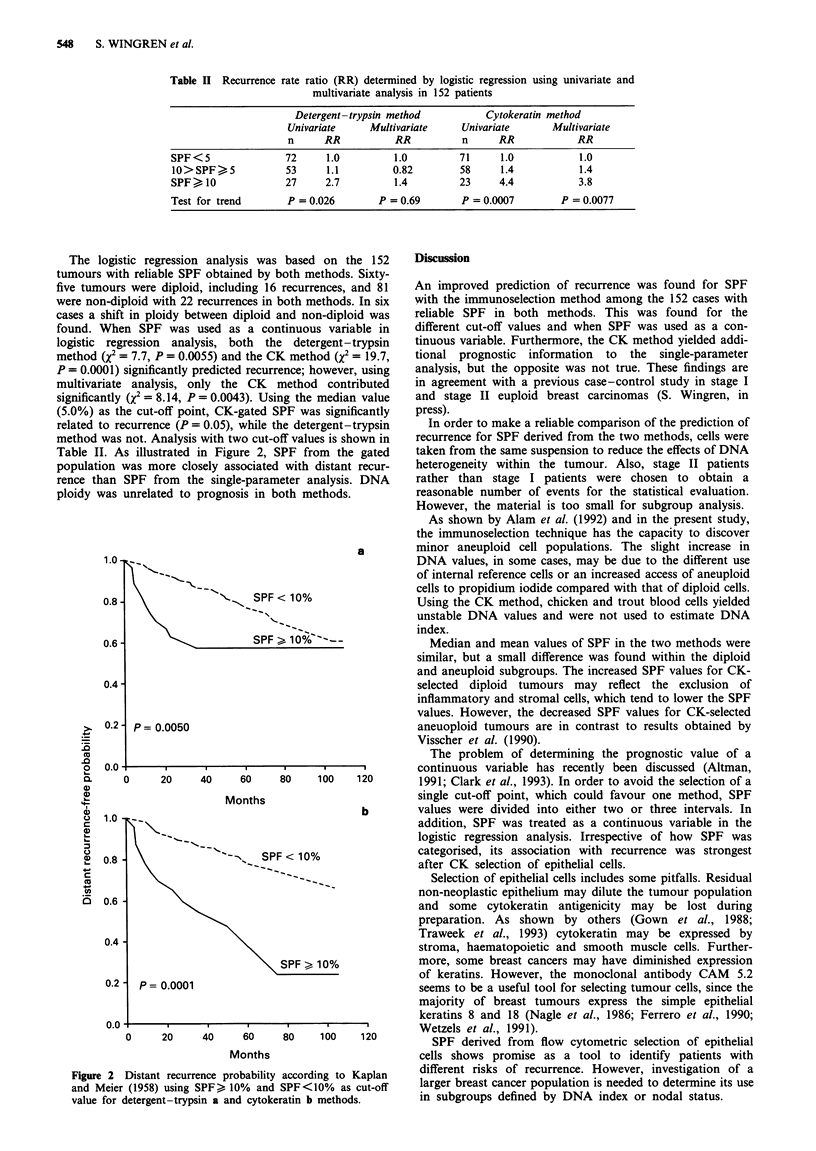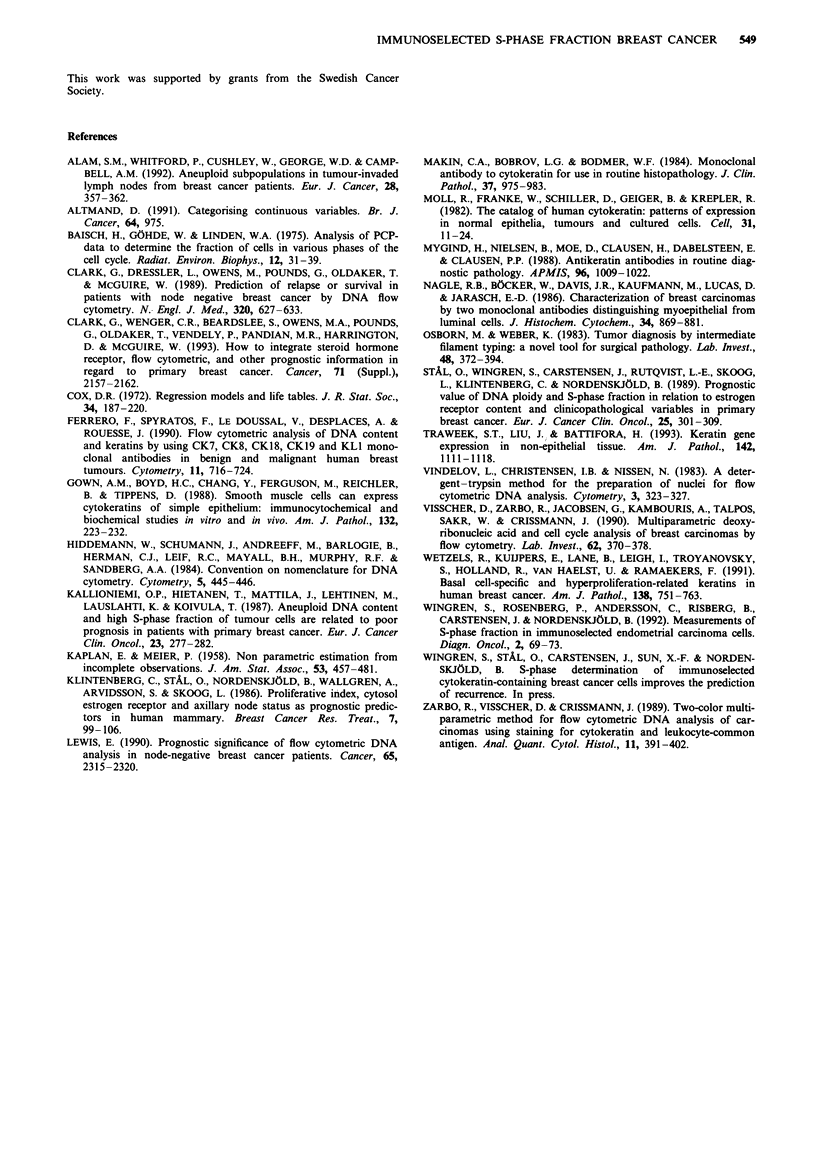# Flow cytometric analysis of S-phase fraction in breast carcinomas using gating on cells containing cytokeratin. South East Sweden Breast Cancer Group.

**DOI:** 10.1038/bjc.1994.99

**Published:** 1994-03

**Authors:** S. Wingren, O. Stål, B. Nordenskjöld

**Affiliations:** Department of Oncology, Faculty of Health Sciences, Linköping University, Sweden.

## Abstract

We investigated distant recurrence and S-phase fraction (SPF), estimated by flow cytometry with and without selection of the epithelial cell population, in 201 stage II breast carcinomas. The tumour tissue was disintegrated mechanically by scissors and one part of the cell suspension was treated with a detergent-trypsin method for single-parameter analysis, and the other part, for immunological selection of epithelial cells, was incubated with a monoclonal antibody (CAM 5.2) recognising cytokeratins 8 and 18 and a secondary fluorescein isothiocyanate-labelled antibody. DNA was stained with propidium iodide. In order to compare the methods, statistical analysis was performed on the 152 tumours with S-phase fraction estimated by both methods. Sixty-five tumours were diploid, 81 were aneuploid and six tumours had different ploidy determined by the two methods. Using univariate regression analysis, SPF of the epithelial cell population predicted recurrence more effectively than SPF from single-parameter analysis. In multivariate regression analysis, SPF of the cytokeratin-containing population added significant prognostic information to the SPF of the non-selected cells. We concluded that the use of flow cytometric selection of epithelial breast carcinoma cells enhances the predictability value of SPF.


Br. J. Cancer (1994), 69, 546-549 © Macmillan Press Ltd., 1994

### Flow cytometric analysis of S-phase fraction in breast carcinomas using gating on cells containing cytokeratin

S. Wingren, O. Stål & B. Nordenskjöld for the South East Sweden Breast Cancer Group

*Department of Oncology, Faculty of Health Sciences, Linköping University, S581 85 Linköping, Sweden.*

**Summary** We investigated distant recurrence and S-phase fraction (SPF), estimated by flow cytometry with and without selection of the epithelial cell population, in 201 stage II breast carcinomas. The tumour tissue was disintegrated mechanically by scissors and one part of the cell suspension was treated with a detergent-trypsin method for single-parameter analysis, and the other part, for immunological selection of epithelial cells, was incubated with a monoclonal antibody (CAM 5.2) recognising cytokeratins 8 and 18 and a secondary fluorescein isothiocyanate-labelled antibody. DNA was stained with propidium iodide. In order to compare the methods, statistical analysis was performed on the 152 tumours with S-phase fraction estimated by both methods. Sixty-five tumours were diploid, 81 were aneuploid and six tumours had different ploidy determined by the two methods. Using univariate regression analysis, SPF of the epithelial cell population predicted recurrence more effectively than SPF from single-parameter analysis. In multivariate regression analysis, SPF of the cytokeratin-containing population added significant prognostic information to the SPF of the non-selected cells. We conclude that the use of flow cytometric selection of epithelial breast carcinoma cells enhances the predictability value of SPF.

DNA ploidy and SPF have, in several studies, shown good predictibility value in breast carcinomas (Klintenberg *et al.*, 1986; Kallioniemi *et al.*, 1987; Clark *et al.*, 1989; Stål *et al.*, 1989; Lewis, 1990). Aneuploidy and high SPF are generally associated with early distant recurrence and decreased survival time, while diploidy and low SPF correlate with good prognosis.

Estimation of SPF in carcinomas using single-parameter flow cytometry is complicated by the content of inflammatory, stromal and normal epithelial cells in the tumour. The risk of underestimating SPF depends on the proportion of diluting host cells (Wingren *et al.*, 1992) and is highly variable from tissue to tissue and within the same type of tissue. It is, thus, impossible to introduce correction factors because of this sample heterogeneity. The contamination of DNA diploid cancer cells by non-neoplastic cells results in an overlap in the diploid region of the histogram and increases the risk of falsely low SPF values. The dilution of aneuploid tumours with host cells decreases the ability to detect minor populations and may introduce artifacts into the calculation of SPF. Cancer tissue with a low proportion of DNA tetraploid cells compared with diploid cells may be misinterpreted as a DNA diploid tumour. Furthermore, overlap of the tetraploid stemline by diploid G2/M cells makes the assessment of SPF unreliable in some cases.

Although these potential pitfalls are numerous, they may be solved using immunocytochemical technology. However, tumour-specific markers are not available for flow cytometric selection of cancer cells; epithelial cells normally express cytokeratins in a tissue-specific fashion, which may be used for identification (Moll *et al.*, 1982). The vast majority of normal and malignant mammary epithelial cells contain cytokeratins 7, 8, 18 and 19. The characteristics of cytokeratin in normal epithelia of the breast are mostly well preserved during malignant progression and, to some extent, even more pronounced in carcinomas (Osborn *et al.*, 1983; Ferrero *et al.*, 1990; Wetzels *et al.*, 1991). A fluorescein isothiocyanate (FITC)-conjugated secondary antibody, together with a primary monoclonal antibody specific for cytokeratin, used with a suspension of cells with preserved antigenicity, allows flow cytometric sorting of the epithelial cell population (Zarbo *et al.*, 1989; Visscher *et al.*, 1990).

We have now compared the ability of SPF measured on unselected cells and SPF estimated on immunoselected epithelial cells to predict recurrence of stage II breast cancer.

Correspondence: S. Wingren.
Received 6 July 1993; and in revised form 8 October 1993.

### Materials and methods

Two hundred and one patients with primary breast cancer in pathological stage II (UICC), operated on between 1977 and 1990, were included in the study. The patients' median age was 57 years and the median follow-up time was 59 months. Seventy per cent of the tumours had oestrogen receptor levels greater than 0.1 fmol per μg of DNA and 35% were 20 mm or less in diameter. Twenty per cent of the patients were lymph node negative, while 53% and 27% had 1-3 and >3 metastatic nodes respectively. Forty-eight patients had distant recurrence during the follow-up period. The tumour samples were kept frozen at −70°C until analysis.

### *Preparation for flow cytometric analysis*

In order to confirm the presence of cancer cells, touch preparations stained with May-Grunewald-Giemsa solutions were used and examined in a light microscope. The frozen tissue was cut with scissors in a citrate buffer before filtration through a nylon mesh (pore size 41 μm). Cell suspensions were divided for preparation into single- and dual-flow cytometric analysis.

The cell suspension used for flow cytometric gating on cytokeratin-containing epithelial cells (CK) was fixed in cold (−20°C) 70% ethanol and stored at 4°C overnight. After centrifugation (890 *g*), 1 ml of a PAB solution containing phosphate-buffered saline with 0.5% serum albumin was added. The primary mouse monoclonal antibody CAM 5.2 (Makin *et al.*, 1984; Mygind *et al.*, 1988), recognising cytokeratin 8 and 18 (Becton Dickinson No. 7650), was incubated for 30 min. The secondary fluorescein isothiocyanate-conjugated monoclonal antibody $F(ab)_2$ (Dakopatts No. F313) was added after washing and resuspension in 1 ml of PAB. The cell suspension was washed twice before resuspension in 600 μl of PAB solution containing RNAse (50 μg $ml^{-1}$). After aspiration with a syringe (needle diameter 0.4 mm) and filtration as described above, DNA was stained with 15 μg of propidium iodide before analysis with flow cytometry.

The cell suspension for single-parameter analysis was prepared as described by Vindelöv *et al.* (1983). Briefly, cells were treated with a detergent trypsin solution before the addition of trypsin inhibitor and spermine tetrahydrochloride. Nuclei were labelled with propidium iodide prior to flow cytometric analysis. Chicken and trout blood cells were used as internal standards to estimate DNA index.

*Flow cytometry*

A FACscan flow cytometer (Becton Dickinson) equipped with a 15 mW argon laser (488 nm) was used. Fifteen thousand events were recorded in a dot plot (cytokeratin *vs* DNA). A window was placed in the area of cytokeratin-positive cells, generating a histogram for the evaluation of SPF of the epithelial population. Histograms with fewer than 1,000 cytokeratin-positive cells were not evaluated. S-phase fraction was calculated assuming a rectangular distribution (Baisch *et al.*, 1975). The number of channels between the G0/G1 and G2/M peaks was multiplied by the mean number of cells per channel in an interval interactively selected in the S-phase region of the histogram. Histograms including a single G0/G1 peak were defined as diploid, while tumours with additional G0/G1 stemlines were classified as non-diploid (Hiddeman *et al.*, 1984).

In single-parameter analysis, 15,000 events were recorded and S-phase values were calculated as described above. Chicken and trout blood cells were used to estimate the DNA index.

All S-phase values were corrected for background by selecting an area to the right of the G2/M peak with a representative amount of debris. The mean counts per channel in this region was subtracted from the mean number of cells in the S-phase area. In order to reduce the number of cell clumps, doublet discrimination was performed on the dot plot of the area and width of the red signal.

*Statistical methods*

The association between SPF and recurrence rate was analysed using the proportional hazards model described by Cox (1972) and recurrence curves were calculated according to Kaplan and Meier (1958).

### Results

SPF was considered reliable in 173 (86%) tumours using the detergent method, and 167 (83%) with the CK method. Mean and median values of SPF are shown in Table I. The mean coefficient of variation (CV) for the detergent–trypsin and cytokeratin methods was 3.95 and 4.11 respectively.

In four cases an additional peak was found as a result of the increased ability of the CK method to identify small aneuploid stemlines. Figure 1 illustrates a non-diploid tumour with and without selection of epithelial cells. However, the cytokeratin method yielded marginally higher DNA values. The difference between the methods increased significantly with increased values of DNA index. Twelve tumours with a DNA index close to the limits of the DNA tetraploid region using the single-parameter analysis were thus candidates to be classified as tetraploid or hypertetraploid with the cytokeratin method. With the hypodiploid tumours, the CK method failed to identify the hypodiploid stemlines in 4 of 10 cases.

**Table I** Distribution of mean and median SPF determined by the cytokeratin method (CK) and detergent–trypsin method (DT) in the 152 patients with SPF by both methods

| | *All tumours* | | *Diploid tumours* | | *Non-diploid tumours* | |
|---|---|---|---|---|---|---|
| | *CK* | *DT* | *CK* | *DT* | *CK* | *DT* |
| Mean | 6.0 | 6.1 | 4.6 | 4.1 | 7.1 | 7.8 |
| Median | 5.2 | 5.0 | 4.0 | 3.7 | 6.2 | 7.0 |

**Figure 1** Dot plots and histograms of a non-diploid tumour before **a** and after **b** selection of cytokeratin-containing cells.

**Table II** Recurrence rate ratio (RR) determined by logistic regression using univariate and multivariate analysis in 152 patients

| | *Detergent–trypsin method* | | | *Cytokeratin method* | | |
|---|---|---|---|---|---|---|
| | *Univariate* | | *Multivariate* | *Univariate* | | *Multivariate* |
| | n | *RR* | *RR* | n | *RR* | *RR* |
| SPF<5 | 72 | 1.0 | 1.0 | 71 | 1.0 | 1.0 |
| 10>SPF⩾5 | 53 | 1.1 | 0.82 | 58 | 1.4 | 1.4 |
| SPF⩾10 | 27 | 2.7 | 1.4 | 23 | 4.4 | 3.8 |
| Test for trend | $P = 0.026$ | | $P = 0.69$ | $P = 0.0007$ | | $P = 0.0077$ |

The logistic regression analysis was based on the 152 tumours with reliable SPF obtained by both methods. Sixty-five tumours were diploid, including 16 recurrences, and 81 were non-diploid with 22 recurrences in both methods. In six cases a shift in ploidy between diploid and non-diploid was found. When SPF was used as a continuous variable in logistic regression analysis, both the detergent–trypsin method ($\chi^2 = 7.7$, $P = 0.0055$) and the CK method ($\chi^2 = 19.7$, $P = 0.0001$) significantly predicted recurrence; however, using multivariate analysis, only the CK method contributed significantly ($\chi^2 = 8.14$, $P = 0.0043$). Using the median value (5.0%) as the cut-off point, CK-gated SPF was significantly related to recurrence ($P = 0.05$), while the detergent–trypsin method was not. Analysis with two cut-off values is shown in Table II. As illustrated in Figure 2, SPF from the gated population was more closely associated with distant recurrence than SPF from the single-parameter analysis. DNA ploidy was unrelated to prognosis in both methods.

**Figure 2** Distant recurrence probability according to Kaplan and Meier (1958) using SPF⩾10% and SPF<10% as cut-off value for detergent–trypsin **a** and cytokeratin **b** methods.

### Discussion

An improved prediction of recurrence was found for SPF with the immunoselection method among the 152 cases with reliable SPF in both methods. This was found for the different cut-off values and when SPF was used as a continuous variable. Furthermore, the CK method yielded additional prognostic information to the single-parameter analysis, but the opposite was not true. These findings are in agreement with a previous case–control study in stage I and stage II euploid breast carcinomas (S. Wingren, in press).

In order to make a reliable comparison of the prediction of recurrence for SPF derived from the two methods, cells were taken from the same suspension to reduce the effects of DNA heterogeneity within the tumour. Also, stage II patients rather than stage I patients were chosen to obtain a reasonable number of events for the statistical evaluation. However, the material is too small for subgroup analysis.

As shown by Alam *et al.* (1992) and in the present study, the immunoselection technique has the capacity to discover minor aneuploid cell populations. The slight increase in DNA values, in some cases, may be due to the different use of internal reference cells or an increased access of aneuploid cells to propidium iodide compared with that of diploid cells. Using the CK method, chicken and trout blood cells yielded unstable DNA values and were not used to estimate DNA index.

Median and mean values of SPF in the two methods were similar, but a small difference was found within the diploid and aneuploid subgroups. The increased SPF values for CK-selected diploid tumours may reflect the exclusion of inflammatory and stromal cells, which tend to lower the SPF values. However, the decreased SPF values for CK-selected aneuoploid tumours are in contrast to results obtained by Visscher *et al.* (1990).

The problem of determining the prognostic value of a continuous variable has recently been discussed (Altman, 1991; Clark *et al.*, 1993). In order to avoid the selection of a single cut-off point, which could favour one method, SPF values were divided into either two or three intervals. In addition, SPF was treated as a continuous variable in the logistic regression analysis. Irrespective of how SPF was categorised, its association with recurrence was strongest after CK selection of epithelial cells.

Selection of epithelial cells includes some pitfalls. Residual non-neoplastic epithelium may dilute the tumour population and some cytokeratin antigenicity may be lost during preparation. As shown by others (Gown *et al.*, 1988; Traweek *et al.*, 1993) cytokeratin may be expressed by stroma, haematopoietic and smooth muscle cells. Furthermore, some breast cancers may have diminished expression of keratins. However, the monoclonal antibody CAM 5.2 seems to be a useful tool for selecting tumour cells, since the majority of breast tumours express the simple epithelial keratins 8 and 18 (Nagle *et al.*, 1986; Ferrero *et al.*, 1990; Wetzels *et al.*, 1991).

SPF derived from flow cytometric selection of epithelial cells shows promise as a tool to identify patients with different risks of recurrence. However, investigation of a larger breast cancer population is needed to determine its use in subgroups defined by DNA index or nodal status.

This work was supported by grants from the Swedish Cancer Society.

### References

ALAM, S.M., WHITFORD, P., CUSHLEY, W., GEORGE, W.D. & CAMPBELL, A.M. (1992). Aneuploid subpopulations in tumour-invaded lymph nodes from breast cancer patients. *Eur. J. Cancer*, **28**, 357–362.

ALTMAND, D. (1991). Categorising continuous variables. *Br. J. Cancer*, **64**, 975.

BAISCH, H., GÖHDE, W. & LINDEN, W.A. (1975). Analysis of PCP-data to determine the fraction of cells in various phases of the cell cycle. *Radiat. Environ. Biophys.*, **12**, 31–39.

CLARK, G., DRESSLER, L., OWENS, M., POUNDS, G., OLDAKER, T. & MCGUIRE, W. (1989). Prediction of relapse or survival in patients with node negative breast cancer by DNA flow cytometry. *N. Engl. J. Med.*, **320**, 627–633.

CLARK, G., WENGER, C.R., BEARDSLEE, S., OWENS, M.A., POUNDS, G., OLDAKER, T., VENDELY, P., PANDIAN, M.R., HARRINGTON, D. & MCGUIRE, W. (1993). How to integrate steroid hormone receptor, flow cytometric, and other prognostic information in regard to primary breast cancer. *Cancer*, **71** (Suppl.), 2157–2162.

COX, D.R. (1972). Regression models and life tables. *J. R. Stat. Soc.*, **34**, 187–220.

FERRERO, F., SPYRATOS, F., LE DOUSSAL, V., DESPLACES, A. & ROUESSE, J. (1990). Flow cytometric analysis of DNA content and keratins by using CK7, CK8, CK18, CK19 and KL1 monoclonal antibodies in benign and malignant human breast tumours. *Cytometry*, **11**, 716–724.

GOWN, A.M., BOYD, H.C., CHANG, Y., FERGUSON, M., REICHLER, B. & TIPPENS, D. (1988). Smooth muscle cells can express cytokeratins of simple epithelium: immunocytochemical and biochemical studies *in vitro* and *in vivo*. *Am. J. Pathol.*, **132**, 223–232.

HIDDEMANN, W., SCHUMANN, J., ANDREEFF, M., BARLOGIE, B., HERMAN, C.J., LEIF, R.C., MAYALL, B.H., MURPHY, R.F. & SANDBERG, A.A. (1984). Convention on nomenclature for DNA cytometry. *Cytometry*, **5**, 445–446.

KALLIONIEMI, O.P., HIETANEN, T., MATTILA, J., LEHTINEN, M., LAUSLAHTI, K. & KOIVULA, T. (1987). Aneuploid DNA content and high S-phase fraction of tumour cells are related to poor prognosis in patients with primary breast cancer. *Eur. J. Cancer Clin. Oncol.*, **23**, 277–282.

KAPLAN, E. & MEIER, P. (1958). Non parametric estimation from incomplete observations. *J. Am. Stat. Assoc.*, **53**, 457–481.

KLINTENBERG, C., STÅL, O., NORDENSKJÖLD, B., WALLGREN, A., ARVIDSSON, S. & SKOOG, L. (1986). Proliferative index, cytosol estrogen receptor and axillary node status as prognostic predictors in human mammary. *Breast Cancer Res. Treat.*, **7**, 99–106.

LEWIS, E. (1990). Prognostic significance of flow cytometric DNA analysis in node-negative breast cancer patients. *Cancer*, **65**, 2315–2320.

MAKIN, C.A., BOBROV, L.G. & BODMER, W.F. (1984). Monoclonal antibody to cytokeratin for use in routine histopathology. *J. Clin. Pathol.*, **37**, 975–983.

MOLL, R., FRANKE, W., SCHILLER, D., GEIGER, B. & KREPLER, R. (1982). The catalog of human cytokeratin: patterns of expression in normal epithelia, tumours and cultured cells. *Cell*, **31**, 11–24.

MYGIND, H., NIELSEN, B., MOE, D., CLAUSEN, H., DABELSTEEN, E. & CLAUSEN, P.P. (1988). Antikeratin antibodies in routine diagnostic pathology. *APMIS*, **96**, 1009–1022.

NAGLE, R.B., BÖCKER, W., DAVIS, J.R., KAUFMANN, M., LUCAS, D. & JARASCH, E.-D. (1986). Characterization of breast carcinomas by two monoclonal antibodies distinguishing myoepithelial from luminal cells. *J. Histochem. Cytochem.*, **34**, 869–881.

OSBORN, M. & WEBER, K. (1983). Tumor diagnosis by intermediate filament typing: a novel tool for surgical pathology. *Lab. Invest.*, **48**, 372–394.

STÅL, O., WINGREN, S., CARSTENSEN, J., RUTQVIST, L.-E., SKOOG, L., KLINTENBERG, C. & NORDENSKJÖLD, B. (1989). Prognostic value of DNA ploidy and S-phase fraction in relation to estrogen receptor content and clinicopathological variables in primary breast cancer. *Eur. J. Cancer Clin. Oncol.*, **25**, 301–309.

TRAWEEK, S.T., LIU, J. & BATTIFORA, H. (1993). Keratin gene expression in non-epithelial tissue. *Am. J. Pathol.*, **142**, 1111–1118.

VINDELOV, L., CHRISTENSEN, I.B. & NISSEN, N. (1983). A detergent–trypsin method for the preparation of nuclei for flow cytometric DNA analysis. *Cytometry*, **3**, 323–327.

VISSCHER, D., ZARBO, R., JACOBSEN, G., KAMBOURIS, A., TALPOS, SAKR, W. & CRISSMANN, J. (1990). Multiparametric deoxyribonucleic acid and cell cycle analysis of breast carcinomas by flow cytometry. *Lab. Invest.*, **62**, 370–378.

WETZELS, R., KUIJPERS, E., LANE, B., LEIGH, I., TROYANOVSKY, S., HOLLAND, R., VAN HAELST, U. & RAMAEKERS, F. (1991). Basal cell-specific and hyperproliferation-related keratins in human breast cancer. *Am. J. Pathol.*, **138**, 751–763.

WINGREN, S., ROSENBERG, P., ANDERSSON, C., RISBERG, B., CARSTENSEN, J. & NORDENSKJÖLD, B. (1992). Measurements of S-phase fraction in immunoselected endometrial carcinoma cells. *Diagn. Oncol.*, **2**, 69–73.

WINGREN, S., STÅL, O., CARSTENSEN, J., SUN, X.-F. & NORDENSKJÖLD, B. S-phase determination of immunoselected cytokeratin-containing breast cancer cells improves the prediction of recurrence. In press.

ZARBO, R., VISSCHER, D. & CRISSMANN, J. (1989). Two-color multiparametric method for flow cytometric DNA analysis of carcinomas using staining for cytokeratin and leukocyte-common antigen. *Anal. Quant. Cytol. Histol.*, **11**, 391–402.